# Roles of abscisic acid in regulating ripening and quality of strawberry, a model non-climacteric fruit

**DOI:** 10.1093/hr/uhac089

**Published:** 2022-04-22

**Authors:** Bai-Jun Li, Donald Grierson, Yanna Shi, Kun-Song Chen

**Affiliations:** 1 College of Agriculture and Biotechnology, Zhejiang University, Zijingang Campus, Hangzhou 310058, China; 2 Zhejiang Provincial Key Laboratory of Horticultural Plant Integrative Biology, Zhejiang University, Zijingang Campus, Hangzhou 310058, China; 3 State Agriculture Ministry Laboratory of Horticultural Plant Growth, Development and Quality Improvement, Zhejiang University, Zijingang Campus, Hangzhou 310058, China; 4 Division of Plant and Crop Sciences, School of Biosciences, University of Nottingham, Sutton Bonington Campus, Loughborough LE12 5RD, UK

## Abstract

Abscisic acid (ABA) is a dominant regulator of ripening and quality in non-climacteric fruits. Strawberry is regarded as a model non-climacteric fruit due to its extensive genetic studies and proven suitability for transgenic approaches to understanding gene function. Strawberry research has contributed to studies on color, flavor development, and fruit softening, and in recent years ABA has been established as a core regulator of strawberry fruit ripening, whereas ethylene plays this role in climacteric fruits. Despite this major difference, several components of the interacting genetic regulatory network in strawberry, such as MADS-box and NAC transcription factors, are similar to those that operate in climacteric fruit. In this review, we summarize recent advances in understanding the role of ABA biosynthesis and signaling and the regulatory network of transcription factors and other phytohormones in strawberry fruit ripening. In addition to providing an update on its ripening, we discuss how strawberry research has helped generate a broader and more comprehensive understanding of the mechanism of non-climacteric fruit ripening and focus attention on the use of strawberry as a model platform for ripening studies.

## Introduction

Many fleshy fruits are important and popular crops worldwide, and their color, softening, flavor, aroma, and other aspects of quality determine commercial value, consumer satisfaction and preference [1, 2]. Mechanisms that determine fleshy fruit quality have therefore received wide attention. Fruit quality attributes, such as color, flavor, texture, and aroma are products of the ripening process, and the formation of these quality attributes is regulated by the ripening process [3]. Fleshy fruits can be divided into climacteric and non-climacteric types, depending on whether or not there is an obvious peak of respiration and ethylene emission during the onset of fruit ripening. Phytohormones act as crucial regulators of quality and ripening, modulating expression of genes that determine quality attributes, but different hormonal ripening mechanisms appear to act in these two types of fruits [4]. Climacteric fruit ripening is dominated by ethylene and its mechanism has been widely studied [5], while abscisic acid (ABA) plays a pivotal role in non-climacteric fruit ripening [3, 6], although it is becoming clear that it also plays an important role in climacteric fruit [7, 8]. While phytohormones are absolutely required regulators of fruit ripening and quality formation, other external and internal factors, such as light, temperature, and transcriptional and epigenetic regulators are also important for the progression and outcome of the ripening process [9–12].

The mechanism of fruit ripening mediated by ethylene has been described comprehensively in tomato (*Solanum* spp.) [7, 13], a model climacteric fruit, and the function of the genes involved in regulating ripening and fruit quality in other climacteric fruits has been verified via this climacteric model platform [14–17]. In contrast, mechanisms determining fruit ripening and quality by ABA have been less well studied in non-climacteric fruits, due to the lack of an efficient transgenic system. Recently, strawberry (*Fragaria* spp.) has been exploited as a model non-climacteric fruit due to the successful development of transient and stable gene expression systems and CRISPR/Cas9 gene editing [18, 19], which facilitate the verification of ripening gene function [20–22]. In addition to providing a model system for non-climacteric fruit ripening, exploring the mechanisms of ripening in strawberry can also provide useful information for improving the quality of strawberry and other fruits.

Strawberries are important fruits worldwide due to their appealing color, taste and nutritional value, especially, the modern cultivated strawberry (*Fragaria* × *ananassa*, an octoploid species), crossbred from two octoploid parents (*Fragaria virginiana* and *F. chiloensis*) [23], and the mechanism of ripening in this octoploid strawberry fruit has become an attractive research hotspot. Additionally, *Fragaria vesca*, a diploid strawberry, is another focus due to its simpler genetics and mature transgenic systems, and much excellent fundamental work on fruit development and quality formation has been reported in this strawberry [24, 25]. The flexible application of these transgenic methods is an efficient strategy to explore mechanisms of octoploid or diploid strawberry fruit ripening. Here, we review recent research on this model non-climacteric fruit, focusing on the role of ABA in regulating ripening and the mechanisms of quality generation. We also discuss results with different types of strawberry and sampling differences (i.e. whether samples contain achenes plus receptacle or receptacle only), which may have affected hormone and gene expression measurements.

## Evidence for the role of ABA in strawberry fruit ripening

**Figure 1:**
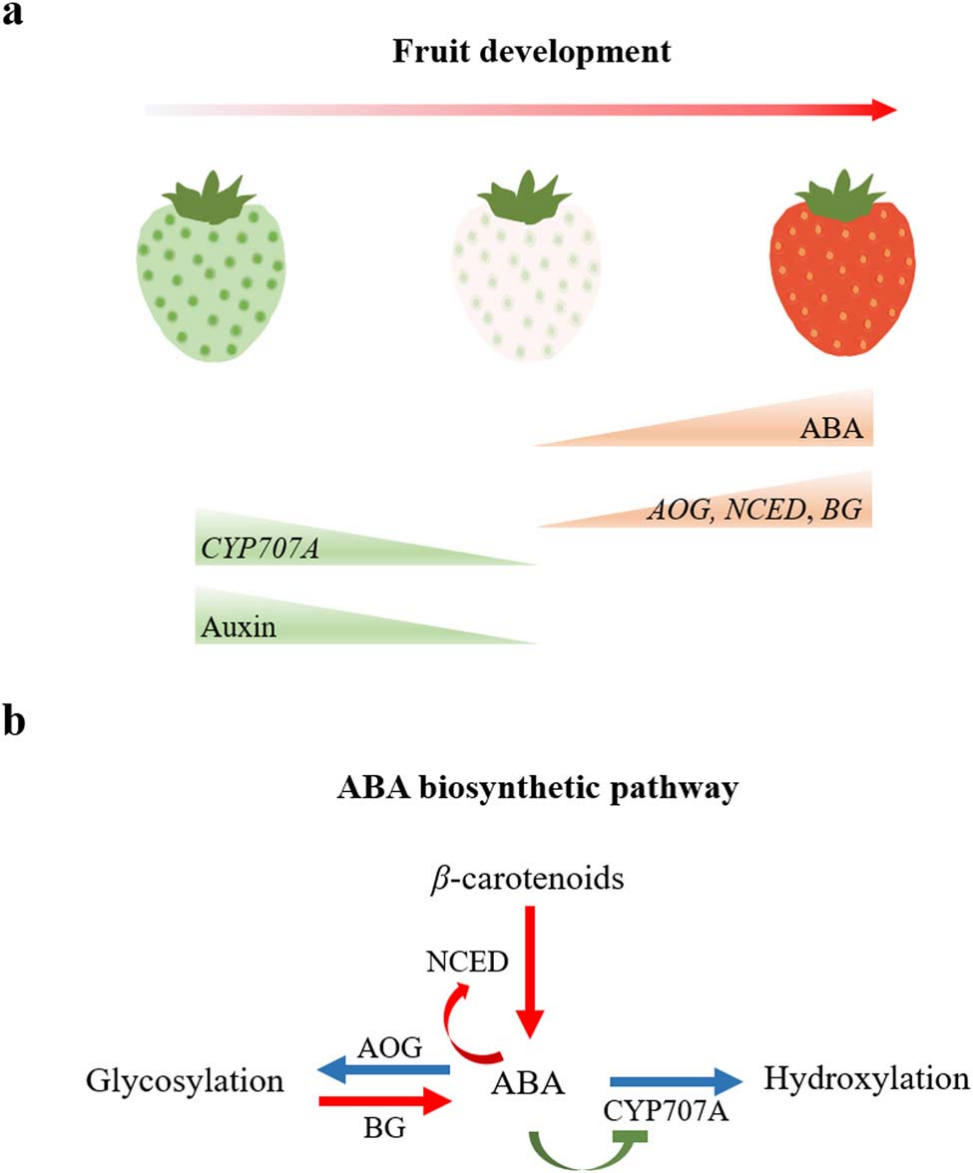
ABA biosynthesis during strawberry fruit development. **a** Changes in transcript levels of the main genes involved in ABA biosynthesis, metabolism, and ABA and auxin content during strawberry fruit development. **b** ABA biosynthetic pathway in strawberry fruit. Red arrows indicate reactions that increase ABA, blue arrows show metabolic reactions reducing active ABA content, and the inhibitory step in green promotes ABA accumulation by inhibiting its metabolism. The *NCED*, *AOG*, *BG*, and *CYP707A* genes have been described [27, 28, 32, 40–42].

The strawberry fruit is a pseudocarp, consisting of a receptacle that is the main edible part, and many achenes (true fruits) embedded in the epidermis of the former [26]. Interestingly, the development of the receptacle depends on auxin produced from the achenes, while ripening relies on ABA, synthesized principally in the receptacle cells [27, 28]. The fruit developmental stages are generally considered to be small green receptacle (SG), large green (LG), degreening (DG), white (WT), initial red (IP), partial red (PR), and full red (FR) according to their color. The increase in ABA content in the receptacle, which is required for strawberry fruit ripening, begins at the WT stage and increases sharply until the FR stage (Fig. 1a) [27–30].

ABA biosynthesis is usually initiated by the cleavage of β-carotenes 9′-*cis*-violaxanthin and 9′-*cis*-neoxanthin, which are converted into xanthoxins by 9-*cis*-epoxycarotenoid dioxygenase (NCED) in plants [31] (Fig. 1b). The expression of *NCED*s gradually increases in parallel with ABA accumulation during strawberry (*Fragaria* spp.) fruit ripening [28, 32]. Furthermore, the ABA content in strawberry (*Fragaria* spp.) fruit is either increased or decreased by *FaNCED1* overexpression or silencing, respectively, which results in promotion or delay of ripening, including quality changes such as sugar accumulation [29], anthocyanin biosynthesis [33], scent production [34], and water conservation [35]. Moreover, the colorless phenotype caused by silencing *FaNCED1* can be rescued by exogenous ABA treatment [29], and an increase in *FveNCED5* expression is necessary for the increase in ABA required for fruit ripening in the *F. vesca* accession ‘Yellow Wonder 5AF7’ (‘YW5AF7’) [28].

The level of active ABA in plant tissues is controlled not only by biosynthesis but also catabolism (Fig. 1b), involving both glycosylation and hydroxylation [36] reactions, catalyzed by abscisate β-glucosyltransferase (AOG), β-d-glucopyranosyl abscisate *β*-glucosidase (BG) [37, 38], and ABA 8′-hydroxylase (CYP707A) [39]. The expression of two *FveAOG*s (*FveUGT71B1* and *FveUGT71B7*) was in accordance with the increase in ABA glucose ester (ABA glycosylation products) and ABA content during *F. vesca* fruit development [27]. Recently, FveUGT71A49 and FveUGT73AC3 have been shown to participate in ABA glycosylation using enzymatic activity assays, and transient overexpression of the former in *F*. × *ananassa* ‘Elsanta’ decreases ABA accumulation [40]. The ABA content was negatively correlated with *FaBG2* and *FaCYP707A1* transcript levels during strawberry (*F*. × *ananassa* ‘Seolhyang’) fruit development, and *FaBG1* expression decreased sharply in light green fruit at the 20 days post-anthesis stage [41]. However, another study showed that *FaBG1* expression increased rapidly, concomitantly with the onset of fruit color development in the strawberry *F*. × *ananassa* ‘Camarosa’, and fruit ripening and ABA accumulation were inhibited after inhibiting *FaBG1* expression using virus-induced gene silencing (VIGS) [42]. Moreover, when *FaBG3* was silenced using VIGS, strawberry (*F*. × *ananassa* ‘Albion’) fruit had lower ABA and sugar contents and were firmer and paler compared with the control, due to the downregulation of several ripening-related genes [43]. Therefore, the members of the BG family, whose expression increases with strawberry fruit development, promote ripening via enhancing ABA accumulation (Fig. 1a). On the other hand, it has been verified that *FveCYP707A4a* acts to prevent ABA accumulation in the fruit during the early stages of growth in ‘YW5AF7’ strawberry, and transient overexpression or knockdown (using RNA interference, RNAi) of *FveCYP707A4a* either enhanced or reduced fruit firmness, respectively [28]. Additionally, ABA accumulation can repress *FveCYP707A4a* expression via an *FveCYP707A4a*-based feedback loop [28] (Fig. 1b). Taken together, these results indicate that strawberry fruit ripening and quality are regulated by the endogenous ABA level, which is determined by the balance between the expression levels of *NCED*s, *AOG*s, *BG*s, and *CYP707A*s (Fig. 1). The feedback and feedforward loops act as an important mechanism for controlling the spatiotemporal biosynthesis of ABA during strawberry fruit development.

## Effects of exogenous ABA and its biosynthetic inhibitor on fruit quality attributes

In addition to the changes in endogenous ABA during fruit development, evidence of its importance in influencing strawberry fruit ripening traits has also come from application of exogenous ABA and its biosynthesis inhibitors. Anthocyanins contribute to the red color of ripe strawberry (*Fragaria* spp.) fruit, and positive regulators of the biosynthetic pathway (anthocyanin biosynthetic pathway, ABP), including *MYB10*s and an *AP2*/*EFR* gene *Fragaria related to ABI3/VP1* (*FaRAV1*), can directly influence anthocyanin accumulation by upregulating ABP structural genes, and these gene transcripts show a positive correlation with ABA content [41, 44, 45]. Exogenous application of ABA promotes anthocyanin accumulation by increasing the expression levels of *MYB10*s, *FaRAV1*, and ABP structural genes in strawberry fruits (*Fragaria* spp.) [33, 45], while the opposite results were found using ABA inhibitor treatments [27, 46, 47].

The contents of sugars and volatile compounds contribute to the sweetness and aroma of strawberry fruits that are valued by consumers [2]. Sucrose is one of the main soluble sugars in strawberry fruits [48], and injecting exogenous ABA into the fruit can boost its production by increasing the expression of *sucrose transporter gene 1* (*FaSUT1*), a sucrose transporter participating in loading sucrose into the sieve element companion cell complex and the distribution of sucrose in plant tissues [49], and *ABA-stress-ripening* (*FaASR*), a transcription factor gene involved in fruit ripening [30, 50]. Cytosolic *glyceraldehyde-3-phosphate dehydrogenase 2* (*FaGAPC2*) and plastid *glyceraldehyde-3-phosphate dehydrogenase 1* (*FaGAPCp1*) have key roles in the glycolysis pathway. These genes negatively regulate strawberry (*F*. × *ananassa* ‘Benihoppe’) fruit ripening, anthocyanin biosynthesis, sugar accumulation, and softening, and their expression was inhibited after spraying fruit with a mixture of ABA and sucrose [51]. 4-Hydroxy-2,5-dimethyl-3(2H)-furanone (HDMF) is one of the major contributors to the aroma of *F*. × *ananassa* fruit. HDMF content is directly controlled by quinone oxidoreductase (FaQR), which is the last enzyme in the HDMF biosynthesis pathway [52], and the transcript level of *FaQR* was increased in *F*. × *ananassa* ‘Benihoppe’ fruit in response to high ABA content [53]. Additionally, eugenol, another important volatile component of strawberry fruit scent, and the expression of its positive biosynthesis regulators, *EMISSION OF BENZENOID II* (*FaEOBII*) and *DNA binding one zinc finger* (*FaDOF2*), could be reduced by application of nordihydroguaiaretic acid (NDGA), an inhibitor of NCED activity, which represses ABA biosynthesis [54], or induced by ABA treatment [34, 55].

Texture change of strawberry fruit during ripening is mainly caused by softening, due to changes in primary cell wall metabolism, which make an important contribution to fruit mouthfeel [56]. The transcript levels of several genes encoding cell-wall-modifying enzymes are correlated with ABA content during strawberry fruit ripening (*F. vesca*) [27]. Molina-Hidalgo *et al*. [57] found that when the pectinase gene *rhamnogalacturonate lyase 1* (*FaRGLyase1*) was transiently targeted by RNAi in strawberry fruit (*F*. × *ananassa* ‘Camarosa’), the cell walls had more pectin, cell-wall integrity was increased, and there were fewer intercellular spaces compared with control fruit. The expression of *FaRGLyase1* declined in the receptacle with NDGA treatment, which suggested that ABA could promote strawberry fruit softening via enhancing *FaRGLyase1* expression. Downregulation of *β-galactosidase 4* (*FaβGal4*) using antisense constructs increased the cell wall galactose levels and reduced pectin solubilization in strawberry (*F*. × *ananassa* ‘Camarosa’) fruit, which were on average 30% firmer than control fruit, and *FaβGal4* expression was also found to be positively regulated by ABA [58]. Additionally, exogenous ABA treatment promoted the expression of *F*. × *ananassa expansin 2* (*FaEXP2*), *β-xylosidase 1* (*FaXyl1*), *xyloglucan endotransglycosylase/hydrolase 1* (*FaXTH1*), and *F. chiloensis xyloglucan endotransglycosylase/hydrolase 1* (*FcXTH1*) in parallel with accelerated fruit softening [59–62].

Expression of some transcription factors and other genes involved in regulating strawberry fruit ripening is influenced by exogenous ABA treatment, which indicates that they are probably also components in the network of ABA-mediated strawberry fruit ripening. *SHATTERPROOF*-like (*FaSHP*), a C-type MADS-box gene, is a positive regulator of aspects of strawberry (*F*. × *ananassa* ‘Elsanta’) fruit ripening, including softening, color, ascorbic acid and aroma production [63]. Expression of *FaSHP* was upregulated significantly after spraying fruit with exogenous ABA at the white receptacle/brown achenes stage, which significantly increased the expression of quality-related genes involved in color (*FaMYB10*, ABP structural genes), softening [*polygalacturonase 1* (*FaPG1*), *pectate lyase* (*FaPL*), *endo-β-1,4-glucanase 1* (*FaEG1*) etc.] and aroma (*FaQR*) [63]. Transiently overexpressing *FaMADS1a*, another MADS-box gene belonging to the SEP1/2 clade, in strawberry (*F*. × *ananassa* ‘Akihime’) fruit could delay the ripening process by inhibiting anthocyanin biosynthesis by repressing the ABP structural genes, and *FaMADS1a* expression was also decreased in exogenous ABA-treated fruit [64]. In addition, the expression level of *Ripening Inducing Factor* (*FaRIF*), encoding an *NAC* transcription factor that is a positive regulator participating in strawberry fruit ripening (*F*. × *ananassa* ‘Camarosa’), was decreased by NDGA treatment of the fruit [65, 66].

The concentration of endogenous ABA in strawberry fruit is clearly determined by the combined effects of ABA biosynthesis, conjugation and metabolism (Fig. 1). In mature strawberry fruit at the ripening stage, high ABA content evokes expression of a series of genes required for the development of quality attributes, including anthocyanin accumulation, flavor formation, and softening. Although there is powerful evidence that ABA content plays a pivotal role in strawberry fruit ripening and quality formation, there are several studies which suggest that ABA application does not promote strawberry ripening [67, 68]. Further research is required to determine the endogenous ABA levels and status of the ABA response systems in these situations. The majority of research reports, however, strongly suggest that exogenous ABA treatment can induce strawberry fruit ripening and the development of fruit quality traits.

## ABA perception and signaling during strawberry ripening

**Figure 2:**
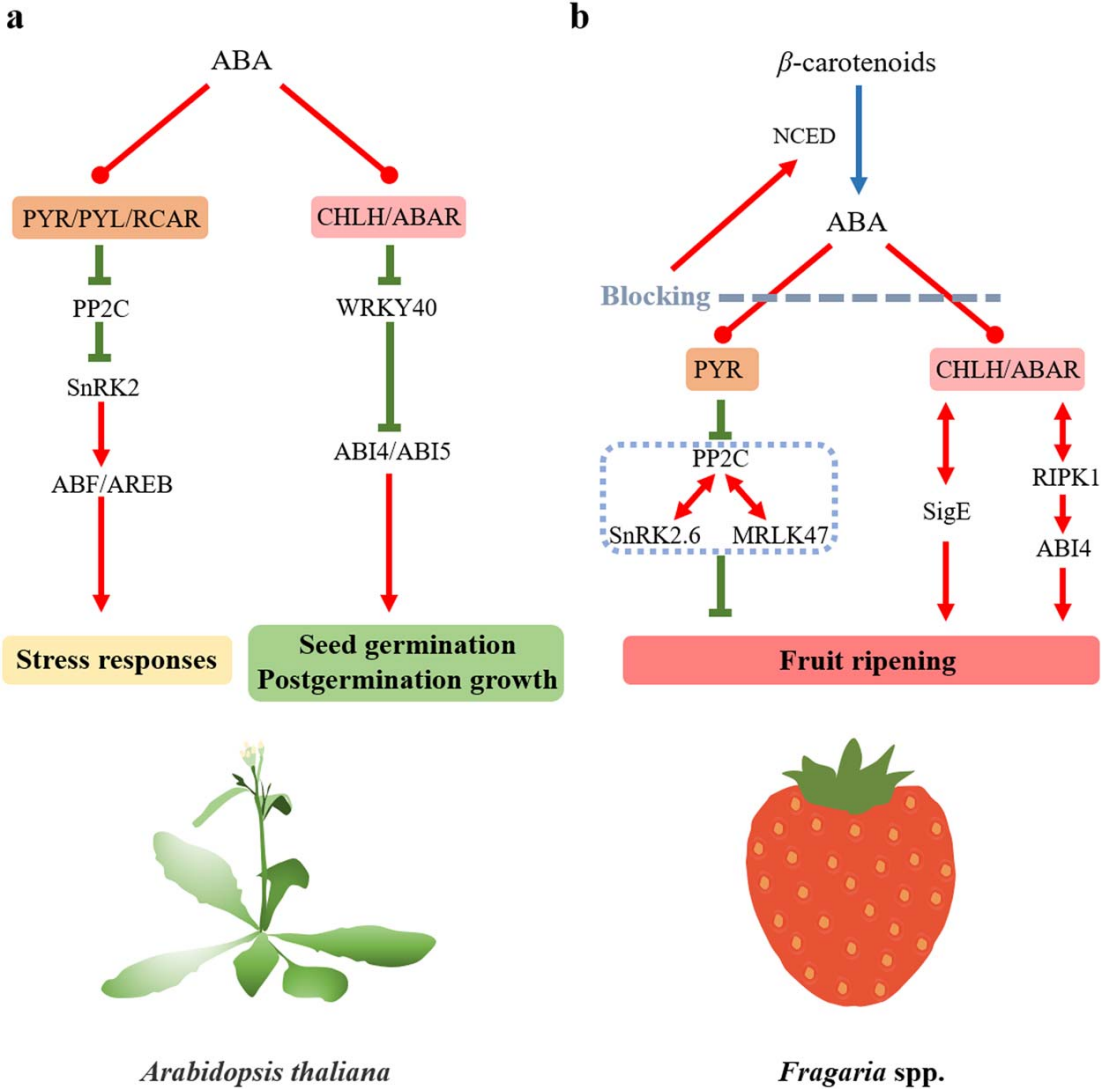
ABA signaling in *A. thaliana* and strawberry fruit. **a** Model of ABA signaling pathway in *A. thaliana*. The PYR/PYL/RCAR-PP2C-SnRK2 signaling module is conserved in land plants [36, 69]. There are 14 *Pyr1* and *Pyl1-Pyl13* genes in the *Arabidopsis* genome. Their proteins change conformation when they perceive ABA and bind to group A type 2 C protein phosphatases (PP2Cs, which negatively regulate ABA signaling. This leads to activation (de-repression) of downstream genes encoding SnRK2 kinases, resulting in the upregulation of transcription factors, ion channels, etc. [70, 71]. **b** ABA signaling pathways involved in strawberry fruit ripening. Based on current reports, there are two ABA signaling pathways, including ABA-PYR-PP2C plus others and ABA-CHLH/ABAR plus others, that participate in regulating strawberry fruit ripening [29, 53, 76–84]. Different SnRK2 kinases can play contrasting roles in the ABA signaling pathway. For example, FaSnRK2.6 negatively regulates strawberry fruit ripening, whereas AtSnRK2s play a positive role in the ABA signaling pathway in *Arabidopsis*. Additionally, when ABA signaling is blocked, a feedback loop is activated, which upregulates NCED expression to enhance ABA accumulation in strawberry fruit. The red and blue arrows and green line terminating in a rectangle represent activation, biosynthesis, and inhibition, respectively. The red double-headed arrows indicate a physical interaction between two proteins. Perception is indicated by a red line terminating in a solid circle. The gray dashed line indicates blocking of the ABA signaling pathway in strawberry fruit. ABA, abscisic acid; NCED, 9-cis-epoxycarotenoid dioxygenase; PYR, pyrabactin resistance; PYL, PYR-like; RCAR, regulatory components of the ABA receptors; PP2C, type 2C protein phosphatases; SnRK2, subfamily 2 of SNF1-related kinases; ABF, ABRE-binding factor; ABRE, ABA responsive element binding protein; CHLH/ABAR, magnesium-PROTOPORPHYRIN IX chelatase H subunit; WRKY40, tryptophan-arginine-lysine-tyrosine 40; ABI4, ABSCISIC ACID-INSENSITIVE 4; ABI5, ABSCISIC ACID-INSENSITIVE 5; MRLK47, FERONIA/FER-like receptor kinase 47; SigE, sigma factor E; RIPK1, red-initial protein kinase 1.

The ABA signaling network plays a vital role in plant responses to stress and alterations in ABA content activate a cascade of actions that regulate downstream gene expression [36, 69]. The ‘gate-latch-lock mechanism’ of ABA perception and signaling consists of the pyrabactin resistance (PYRs)/PYR-like (PYLs) regulatory components of the ABA receptors (RCARs), type 2C protein phosphatases (PP2Cs), and subfamily 2 SNF1-related kinases (SnRK2s). ABA binding to its receptors (PYR/PYL/RCARs) enables them to combine with PP2Cs, which results in removal of PP2C repression of SnRK2 activity [70, 71]. The activated SnRK2s can phosphorylate downstream effectors to regulate physiological responses, while PP2Cs interact with SnRK2s to block ABA signaling in the absence of ABA [72–74] (Fig. 2a). In addition, other ABA signaling network components have been described in plants, comprising plastid/chloroplast ABA receptors (magnesium-protoporphyrin IX chelatase H subunits, CHLH/ABARs), tryptophan-arginine-lysine-tyrosine 40 (WRKY40), and ABI4/ABI5s (APETALA2 domain transcription factor/basic leucine zipper transcription factors), called the ABAR-WRKY40-ABI4/ABI5 signaling model, in which ABAR combines with WRKY40s at high ABA levels to remove the repression of the ABI4/ABI5 expression and activate downstream genes [75] (Fig. 2a).

A similar cascade is believed to be involved in ABA signaling during strawberry ripening and this exerts a major influence on regulating the development of quality attributes [76]. In support of the PYR1-PP2C-SnRK2 model, when the expression of the ABA receptor gene *FaPYR1* was repressed in *F*. × *ananassa* ‘Fugilia’ fruit using VIGS, the fruits were pale in color and other aspects of ripening were inhibited, which might result from blocking the expression of other ABA signaling components, including *FaABI1*, *FaABI3*, *FaABI4*, *FaABI5*, and *FaSnRK2* [77]. Additionally, the higher ABA content in the *FaPYR1*-silenced fruit suggested that a feedback loop mediated by *FaPYR1* might exist and be activated after blocking the ABA signaling pathway [77]. *ABSCISIC ACID-INSENSITIVE 1* (*FaABI1*) is a negative regulator of strawberry (*F*. × *ananassa* ‘Camarosa’) fruit ripening and encodes a PP2C that is decreasingly expressed as fruit development proceeds [78]. *FaABI1*-silenced fruit produced by VIGS showed higher anthocyanin accumulation, increased soluble solids contents, and greater softening, which resulted from increases in transcripts of genes, including the ABP biosynthetic genes and *FaPG*, *FaPL1*, *FaSnRK2*, while the opposite results were observed in fruit transiently overexpressing *FaABI1* [78]. Moreover, the ABA content in *FaABI1*-overexpressing (OE-*FaABI1*) fruit was elevated due to the increase of *FaNCED1* expression [78], which supported the suggestion of an ABA feedback loop found by Chai *et al*. [77]. A recent study using strawberry transcriptome and yeast two-hybrid analyses further indicated that FaABI1 could interact with FaPYL2, which might play a major role in ripening [79]. Interestingly, FERONIA/FER-like receptor kinase 47 (FaMRLK47), a negative regulator of ripening and sucrose biosynthesis in strawberry (*F*. × *ananassa* ‘Benihoppe’) fruit, could physically interact with FaABI1 to repress fruit ripening, indicating the participation of additional components in the ABA signaling process in fruit ripening [80] (Fig. 2b). As the last step in the PYR1-PP2C-SnRK2 model (Fig. 2b), SnRK2 plays an important role in fruit ripening. Han *et al*. [53] identified strawberry (*F*. × *ananassa* ‘Benihoppe’) *FaSnRK2.6* and transient silencing using RNA interference (RNAi) and overexpression of *FaSnRK2.6* verified that in fruit it functions as a negative regulator of ripening. It was shown that accumulation of its transcripts could repress anthocyanin biosynthesis, softening, and aroma metabolism, but had no significant effect on the sugar and total titratable acid content. *FaSnRK2.6* expression decreases with strawberry fruit development and its expression is reduced in fruit treated with high exogenous ABA [53]. This strongly suggests that ABA-regulated fruit ripening occurs mainly via the transcriptional regulation of *FaSnRK2.6*, although FaSnRK2.6 could also be capable of physically interacting with FaABI1 [53]. These results indicate either that differences may exist in the ABA-regulated PYR-PP2C-SnRK2 pathway in different plants or organs, or that not all the regulatory components involved in ABA perception and signaling have been identified yet (Fig. 2).

It has been shown the FaCHLH/ABAR (FaABAR) receptor system can bind ABA [81] and that downregulation of *FaABAR* in *F*. × *ananassa* ‘Fugilia’ fruit using VIGS inhibited ripening and induced an anthocyanin-free white phenotype [29]. Sugar content was also lower, possibly caused by increasing *sigma factor E* (*FaSigE*) and *α-amylase* (*FaAMY*) and decreasing *chalcone synthase* (*FaCHS*) expression [29]. Further, *FaSigE*-RNAi strawberry (*F*. × *ananassa* ‘Hongyan’) fruit showed higher firmness and lower anthocyanin, sugar, and ABA contents. Firefly luciferase complementation suggested that FaSigE could interact with FaABAR, indicating that ABA-FaABAR-FaSigE signaling might participate in fruit ripening control [81] (Fig. 2b). Moreover, results obtained with the yeast two-hybrid assay indicated that red-initial protein kinase 1 (FaRIPK1), a leu-rich repeat receptor-like protein kinase, identified from *F*. × *ananassa* ‘Beinongxiang’, could interact with FaABAR, and FaRIPK1 was regarded as a co-receptor with FaABAR to synergistically regulate fruit ripening [82]. Additionally, downregulation of the positive regulator of ripening, *FaRIPK1*, could promote *FaNCED1*, *FaABI4*, and *FaABAR* expression and ABA accumulation [82], which is similar to the result obtained with *FaABAR*-silenced fruit [29], again suggesting that a feedback mechanism mediated by ABA signaling regulates ABA biosynthesis in strawberry fruit. Chai and Shen [83] identified an *FaABI4* from *F*. × *ananassa* ‘Beinongxiang’ that, when silenced using VIGS, reduced sugar content and color and produced fruit with firmer texture, which suggested that *FaABI4* is a positive regulator of strawberry fruit ripening. *FaABI4* expression is upregulated in *RIPK1*-VIGS strawberry fruit, supporting a proposed ABA-FaABAR-FaRIPK1-ABI4 model for control of ripening [82] (Fig. 2b). The expression of *FaWRKY40* showed the highest level in strawberry (*F*. × *ananassa* ‘Camarosa’) fruit at the green stage and this was drastically diminished thereafter [84], which is consistent with its proposed role as a negative regulator that blocks ABA signaling [75]. Additionally, WRKY binding sites have been found in the promoters of several genes involved in strawberry fruit cell wall metabolism [59, 61, 85]. Weighted gene co-expression network analysis (WGCNA) indicated that *FveWRKY* (FvH4_6g42870.1) is co-expressed with the key structural genes involved in ester synthesis during strawberry (*F. vesca* accessions ‘Hawaii4’ and ‘Ruegen’) fruit development and has therefore been suggested to participate in fruit ripening regulation [86]. However, no study has specifically identified *WRKY* and *ABI5* members that participate in regulating strawberry ripening and quality development. Thus, at present, mechanisms of strawberry fruit ripening mediated by the suggested ABAR-WRKY40-ABI4/ABI5 pathway are poorly understood compared with the PYR1-PP2C-SnRK2 pathway. Furthermore, the proposed ABA-FaABAR-FaRIPK1-FaABI4 and ABA-FaABAR-FaSigE pathways for regulating strawberry fruit ripening [82] (Fig. 2b) are different from the ABAR-WRKY40-ABI4/ABI5 stress response pathway in *Arabidopsis thaliana* [70, 71] (Fig. 2a).

Although both ABA signaling pathways appear to operate in the regulation of strawberry fruit ripening, their specific functions and contributions to the development of fruit quality are still unclear. This raises the following questions. (i) How, and to what extent, do these two systems regulate expression of genes related to quality and is this by direct or indirect action? (ii) Do the differences between these pathways and the *A. thaliana* stress response pathway indicate novel or modified ABA signaling mechanisms that may mediate strawberry fruit ripening? These questions need to be explored further in order to achieve a better understanding of how ABA regulates strawberry fruit ripening and quality.

## Transcription factors and epigenetic modifications involved in regulating ABA biosynthesis and signaling

*MADS-box* and *NAC* genes are important regulators of ripening in climacteric fruits [12, 87] and also play a role in non-climacteric fruits such as strawberry [65, 88]. They generally participate in regulating fruit ripening through influencing other ripening genes [12] and phytohormone biosynthesis, especially ethylene and ABA [89–91]. *FaSHP* [63], *FaMADS1a* [64], and *FaMADS9* [88, 92], an ortholog of tomato *Ripening Inhibitor* (*RIN*), have been found to participate in strawberry fruit ripening. The ABA levels of *FaMADS9*-silenced strawberry (*F*. × *ananassa* ‘Camarosa’) fruit generated using stable RNAi were reduced by between 22 and 49% in different transgenic lines, and *FaNCED1/2/3* expression was reduced by >70% and *FaSnRK2.6* was upregulated by >40%, which suggested that *FaMADS9* played a positive role in the ABA pathway of strawberry fruit ripening [92]. Recently, an NAC transcription factor, FaRIF, has been characterized as a positive regulator that promotes ripening, including softening, sucrose accumulation, and coloration of strawberry (*F*. × *ananassa* ‘Camarosa’) fruit using stable RNAi and overexpression assays [65]. Additionally, the ABA content was decreased by 16–25% in *35Spro*:*RIF*-RNAi-treated red receptacles compared with that of control fruit. The induction of *FaNCED3* and *zeaxanthin epoxidase* (*FaZEP*), key genes for carotenoid biosynthesis, and the delayed maturity phenotype of *35Spro*:*RIF*-RNAi fruit could be recovered by infiltrating exogenous ABA. This supports the suggestion that *FaRIF* controls the fruit ripening-related processes by regulating ABA biosynthesis in strawberry fruit [65], although it is possible that it also regulates other genes.

**Figure 3:**
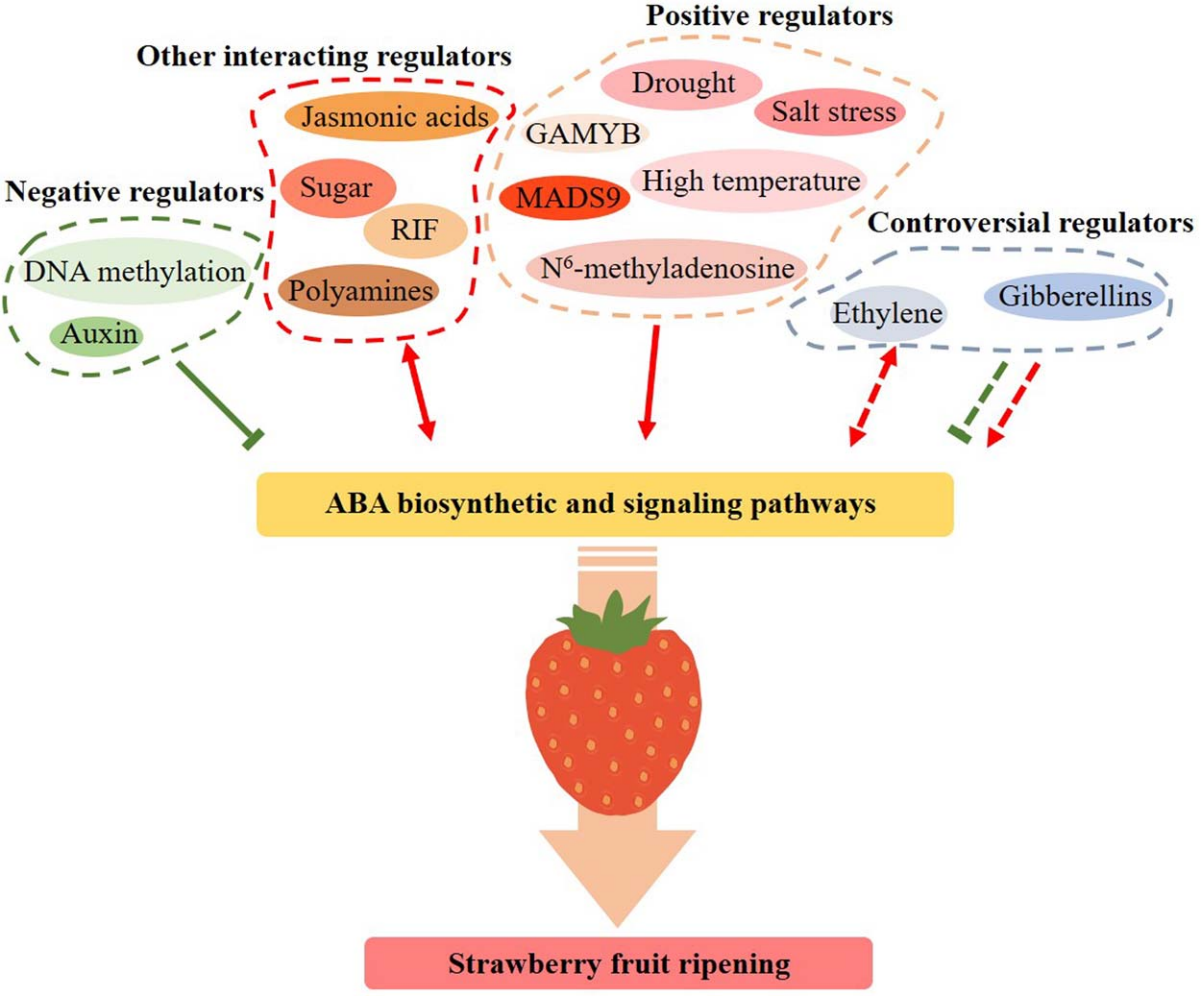
Interplay between ABA and other factors in strawberry fruit ripening. Several endogenous and environmental factors participate in regulating strawberry fruit ripening by influencing ABA biosynthetic and signaling pathways, including negative, positive, other interacting regulators, and some controversial factors, where the evidence is not conclusive. Red arrows indicate positive-acting factors and green lines indicate inhibitory actions. Dashed lines indicate controversial potential regulators, such as ethylene and GA. RIF, ripening inducing factor; GAMYB, GA-responsive MYB transcription factor; MADS9, MADS-box gene 9.

Over the past few years, more and more studies have shown that epigenetic modifications can regulate fruit ripening and quality by controlling expression of related genes [10, 93, 94]. Changes in the level of DNA methylation modulate gene expression [93]. The RNA-directed DNA methylation (RdDM) pathway [10], which decreases during strawberry (*F*. × *ananassa* ‘Hongjia’) fruit ripening, and the action of demethylases, are the main factors that determine DNA methylation [1]. Ripening-induced DNA hypomethylation caused upregulation of the genes related to ABA biosynthesis and quality formation, which suggests that RdDM-mediated DNA demethylation might enhance strawberry fruit ripening by increasing ABA biosynthesis [1]. Methylation of transcripts (mRNAs) is another important epigenetic modification, which occurs post-transcriptionally [94]. Recently, Zhou *et al*. [95] found that mRNAs encoding adenosine methyltransferases, MTA and MTB, were required for strawberry (*Fragaria* spp.) fruit ripening. MTA-mediated N^6^-Methyladenosine (m^6^A) modification enhanced the stability of *NCED5* and *ABA-responsive element-binding protein 1* (*AREB1*) mRNAs, and facilitated the translation of *ABAR*, which suggested that m^6^A modification regulated strawberry fruit ripening by targeting the ABA pathway [95]. This evidence suggests that transcriptional regulation and epigenetic modification are crucial components of the mechanism of strawberry fruit ripening mediated by ABA (Fig. 3).

## Interactions between ABA and other phytohormones

As crucial regulators of plant growth and development, interactions between phytohormones occur frequently. Such interactions, often referred to as ‘crosstalk’, without defining any specific mechanisms, have been reported widely [12, 96–98], e.g. between ABA and other phytohormones, including auxins, gibberellins (GAs), ethylene, and jasmonic acids (JAs) [3, 4, 7].

The early growth of the strawberry receptacle depends on auxins produced from the achenes. The auxin content in the receptacle is higher at the early stage and decreases at later stages of fruit development, while the change in ABA content shows the opposite trend [67, 99–101] (Fig. 1). Previous studies have proposed that the ratio of auxins/ABA is crucially important for the process of strawberry fruit ripening [102, 103]. Application of exogenous auxin delays strawberry fruit ripening and inhibits or promotes expression of *NCED*s or *CYP707A*s, respectively, which arrests ABA accumulation and blocks the ABA signaling pathway by downregulating the expression of *FaPYR1*. This also represses genes required for development of quality attributes, including *MYB10*s, *FaASR*, *FaSHP*, ABP structural genes, *FaQR*, and cell-wall-associated genes, etc. [28, 30, 46, 59, 63, 104, 105]. Moreover, genes related to auxin accumulation and response, including *auxin transporter* (*FaPIN*) and *flavin monooxygenase* (*FaYUCCA*), decline in strawberry fruit in response to exogenous ABA treatment [30]. Recently, it was shown that auxin deactivation genes, including *FveUGT74E1b* and *auxin responsive GH3.1* (*FveGH3.1*), and *small auxin-up RNA* (*FveSAUR*), an auxin-responsive gene, were upregulated in strawberry fruit following NDGA application, while the expression of *FvePILS5*, a *PIN*-like gene [106], was downregulated, resulting in auxin redistribution in cells [27]. Moreover, previous studies showed that removing the achenes from the receptacles of mature unripe fruit (de-achened fruit) could promote ripening, including chlorophyll degradation, anthocyanin biosynthesis, and softening, while the application of auxin could reverse these phenotypes [27, 107, 108]. Additionally, Li *et al*. [43] found that *FaNCED* and *FaCYP707A* were upregulated in de-achened fruit, while exogenous auxin treatment could restore their expression levels. Taken together, these results suggest that auxin produced from achenes and ABA biosynthesized by cells of the receptacles interact to manage the progress of strawberry fruit ripening. The antagonistic action between ABA and auxins in strawberry fruit means a higher auxin content impedes ABA accumulation by downregulating biosynthetic gene expression and blocking the ABA signaling pathway to delay ripening, while auxin biosynthesis, enhanced concentration and signaling may be negatively impacted by higher ABA concentrations (Fig. 3). Further work is necessary to reveal the mechanisms underlying this phytohormone crosstalk.

Transcriptome analysis suggests that GAs are mainly produced from strawberry fruit achenes. They are known to induce parthenocarpy and together with auxins are involved in regulating *F. vesca* fruit development at the early stage [99, 101, 109]. However, there is a controversy about the roles of GAs in strawberry (*Fragaria* spp.) fruit ripening. Several studies have suggested that GAs could positively regulate fruit ripening [84, 110, 111], while others support the suggestion that GA either delays [27, 28, 112] or plays no role in strawberry fruit ripening [67, 103]. Moreover, there is also disagreement about the interaction between ABA and GAs in influencing strawberry ripening. Liao *et al*. [28] proposed that auxin promoted GA biosynthesis and signaling, which enhanced *FveCYP707A4a* expression to inhibit ABA accumulation and strawberry fruit ripening. This would suggest regulation of *FveCYP707A4a* could be a focus for auxin, GA, and ABA interactions during strawberry fruit development. On the other hand, the ripening-related gene *FaGAMYB*, a GA-responsive MYB transcription factor [113], could be upregulated by GAs and promote ABA accumulation through increasing *FaNCED*s expression, which enhances color development of strawberry fruit, hence connecting the GA and ABA signaling pathways during ripening [84]. Thus, there are several possible interactions between GAs and ABA in strawberry fruit ripening, but the role of GA is unclear and requires further exploration (Fig. 3).

Ethylene is a dominant hormone regulating climacteric fruit ripening, especially in tomato and, together with MADS-RIN, is required for the initiation and progression of full ripening [5, 12]. It has also been suggested that there are different regulatory loops that function to control ethylene production, with three slightly different mechanisms operating in different groups of climacteric fruits [114]. ABA can also enhance climacteric fruit ripening and promote coloration (carotenoid production), softening, and formation of aroma and flavor by positively regulating ethylene biosynthesis [7, 13]. Unlike climacteric fruits, however, there is a divergence of opinion about whether ethylene does [115–121] or does not play a regulatory role in strawberry fruit ripening [30, 67, 103, 107, 122, 123]. In postharvest strawberry (*F*. × *ananassa* ‘Sonata’) fruit, exposure to ethylene promotes ABA accumulation, malic acid catabolism, sucrose decline, and weight loss in the receptacle, which suggested that there is an interplay between ethylene and ABA to regulate the progress of strawberry fruit postharvest ripening and senescence [124]. Additionally, silencing *FaBG3* using VIGS decreased ABA accumulation and ethylene emission simultaneously in the strawberry fruit. This increased the expression of *FaETR2* (encoding an ethylene receptor related to ethylene response) and *FaACS1* (encoding the ethylene biosynthetic enzyme 1-aminocyclopropane-1-carboxylic acid synthase 1), while *FaACO2* (*1-aminocyclopropane-1-carboxylic acid oxidase 2*) was downregulated [43]. This result indicates that the ABA level may influence ethylene biosynthesis and or signaling in strawberry fruit. Jiang and Joyce [115] found that ethylene biosynthesis was stimulated in harvested strawberry (*F*. × *ananassa* ‘Everest’) fruit after treating with exogenous ABA and the expression of *FaRIPK1*, a positive regulator of strawberry fruit ripening, could be induced by both ABA and ethylene [82]. In summary, although there are some indications that an interaction between ABA and ethylene may occur during strawberry fruit ripening, the functions of ethylene and its possible interplay with ABA require further investigation and clarification (Fig. 3).

JAs have also been found to positively regulate strawberry (*Fragaria* spp.) fruit ripening and development of quality attributes, including anthocyanin accumulation, sucrose biosynthesis, softening, soluble solids content, and titratable acidity ratio [85, 125–127]. There is also a tight relationship between JAs and ABA during strawberry fruit ripening. The application of methyl jasmonate (MeJA) increased the expression of *FaBG3* in strawberry fruit (*F*. × *ananassa* ‘Fugilia’), whereas ABA application could upregulate *12-oxo-phytodienoic acid* (*FaOPDA1*), a JA biosynthesis gene [30]. Additionally, the application of both ABA and MeJA independently could promote the expression of the same quality-related genes associated with anthocyanin biosynthesis (such as *FaCHS*), cell wall changes (such as *FaXTH1*), and sugar accumulation (such as *FaSUT1*) [29, 30]. Moreover, MeJA application can enhance *FaNCED1*/*2*/*3* expression in *F*. × *ananassa* ‘Benihoppe’ strawberry fruit [127], which is a likely point of interaction between ABA and JA (Fig. 3). However, MeJA application to nutrient solution of WT stage strawberry (*F*. × *ananassa* ‘Aromas’) grown *in vitro* represses *FaNCED1* expression and ABA accumulation on the fifth day of MeJA incubation [128]. This contradicts the conclusion that there is a synergistic effect between JAs and ABA in stimulation and requires further investigation since many other studies indicate that a synergistic relationship does exist.

The key point is that other phytohormones influence ABA levels and affect strawberry fruit ripening and the development of quality attributes. It is important to explore further these interactions at the molecular level to obtain a comprehensive understanding of how non-climacteric fruit ripening is influenced by interactions between ABA and other phytohormones.

**Table 1:** Differences in materials used for studying strawberry fruit ripening mediated by ABA.

$\includegraphics{\bwartpath uhac089t1}$

Fruit samples contained achenes and receptacle. Receptacle samples had achenes removed prior to further experiments or analyses.

## Other metabolites influencing ABA level

In addition to endogenous phytohormones, other endogenous substances can also have an effect on ABA biosynthesis in strawberry. The exogenous application of sucrose significantly promotes expression of *FaNCED*s, while treatment with glucose dramatically increased the expression of *FaBG*s, meaning that sugars can in turn influence ABA accumulation [129]. Transient RNAi silencing or overexpression of *FaSUT1* could also induce up- or downregulation of *FaNCED1* in the fruit, resulting in increased or decreased ABA content, respectively [129]. Additionally, Luo *et al*. [68] found that *FaNCED1* and *FaNCED2* expression was upregulated several days after exogenous sucrose application, which would be expected to promote ABA accumulation and the ripening of strawberry (*F*. × *ananassa* ‘Benihoppe’) fruit. These results suggest that there is an interplay, or crosstalk mechanism, operating between ABA and sugars involved in strawberry fruit ripening (Fig. 3).

Polyamines (PAs) are positively charged biogenic amines that participate in a variety of physiological and developmental processes in plants [130]. Guo *et al*. [120] found that spermine could promote strawberry (*F*. × *ananassa* ‘Sweet Charlie’) fruit ripening, including coloration, softening, and sugar accumulation. Downregulation using VIGS, or overexpression, of *S-adenosyl-L-Met decarboxylase* (*FaSAMDC*), a rate-limiting gene for spermine biosynthesis, could inhibit or enhance *FaNCED1* expression >80% compared with that in control fruits, which resulted in lower or higher ABA accumulation [120]. Furthermore, application of exogenous spermine or the SAMDC inhibitor guanyl hydrazine either promoted or impeded ABA biosynthesis in strawberry fruit, respectively [120]. Additionally, a PA catabolism gene, *polyamine oxidase 5* (*FaPAO5*), has been verified to impede spermine/spermidine biosynthesis and strawberry fruit (*F*. × *ananassa* ‘Zhangji’) ripening, and its expression can be inhibited or upregulated by exogenous ABA or fluridone (an ABA biosynthesis inhibitor) [131]. Moreover, transient overexpression and RNAi experiments showed that a high expression level of *FaPAO5* could reduce the ABA content and the levels of *FaNCED1* and *FaSnRK2.6* transcripts [131]. Therefore, based on current studies, a positive interaction between spermine/spermidine and ABA appears to promote strawberry fruit ripening (Fig. 3).

## Environmental regulators of ABA in strawberry fruits

In plants, ABA is usually associated with responses to stresses, including salinity and especially drought stress [36], which promotes expression of a series of ABA biosynthesis genes, including *FaNCED1*, *aldehyde oxidase* (*FaAAO*), and *FaBG*s, leading to a rise in ABA accumulation and anthocyanin-related genes in the strawberry (*F*. × *ananassa* ‘Camarosa’) fruit, without affecting the fruit yield [132]. Salt stress is a common problem for crop production and can influence fruit quality. Even mild salt stress can elevate the expression of *FaNCED1*, *FaBG3*, *FaEXP*s, *FaPG*, and ABP structural genes, which increases ABA accumulation, softening, and anthocyanin biosynthesis and promotes maturation of the strawberry (*F*. × *ananassa* ‘Camarosa’) fruit [133]. Additionally, temperature is another external factor that can influence fruit ripening and quality formation [134–136]. High and low temperatures suppress and induce *FaSnRK2.6* expression to block and activate ABA signaling, respectively, which can influence strawberry fruit ripening and expression of *FaQR*, *pectinesterase* (*FaPE*), and ABP structural genes that affect fruit quality attributes [53]. Thus, drought, salt stress, and high temperature promote fruit ripening by positively regulating ABA biosynthesis or signaling pathways (Fig. 3).

## Roles of ABA in ripening of postharvest strawberry fruits

Postharvest ripening and senescence are very important for fruit quality, shelf-life, and economic value of fruit on the market [137, 138]. After harvest, strawberry fruits undergo senescence and are susceptible to influences from external factors (mechanical damage and deterioration due to bacterial and fungal infection), which are a common cause of decline in quality during over-maturation [139]. The ABA content of strawberry (*F*. × *ananassa* ‘Akihime’) fruit can increase after harvest to a higher level than in fruit that remain attached to the plant, which may accelerate over-maturation of the harvested fruit [140]. Also, the results from application of exogenous ABA and its biosynthesis inhibitor NDGA also support the proposal that ABA plays a positive role in over-maturation and senescence in postharvest strawberry fruit, as does the antagonistic interplay between ABA and auxins [141, 142]. Water loss by postharvest strawberry (*F*. × *ananassa*) fruit can also enhance ABA accumulation by increasing *FaNCED1* expression [143], which is consistent with other findings that dehydration stress can promote ABA biosynthesis and fruit quality [115]. According to these previous studies, the drought stress caused by dehydration is the major contributor to elevating ABA level rapidly in postharvest strawberry fruit, leading to over-maturation, quality losses, and even deterioration. Therefore, retaining moisture, e.g. by controlling environmental humidity or retaining the carpopodium, to avoid a rise in ABA levels due to water loss, could be an important topic for strawberry postharvest research. Water conservation is important for improving strawberry fruit shelf-life and this is a meaningful research direction that needs more attention and exploration. Further examination of the molecular mechanisms of ABA biosynthesis and signaling in postharvest strawberry fruit is another important field for future research and can provide fundamental information with applied value for improving fruit quality and extending postharvest shelf-life while retaining quality.

## Conclusions and perspective

ABA is a dominant positive regulator that operates at the core of a complex regulatory network governing strawberry fruit ripening and development of quality attributes. The complex mechanism of interacting components involves other phytohormones, environmental factors, transcription factors, and epigenetic modifications that influence expression of ABA biosynthesis genes, *NCED*s, *BG*s, and *CYP707A*s, and ABA signaling components (Fig. 3). This network culminates in the expression of genes that modify color, texture, flavor, and aroma, which generate quality attributes during ripening. Conflicting results in the literature, especially with *F*. × *ananassa*, may be explained by the complicated ploidy of allopolyploid species that cause differences between genomes of cultivars. The presence or absence of achenes, and whether or not they are removed from the receptacle during sampling, may also be of critical importance (Table 1). In summary, the central mechanism of fruit ripening mediated by ABA is nevertheless clear and provides a basic framework for improving strawberry fruit quality by manipulating ABA synthesis and signaling. The use of transient or stable genetic modification tools, including VIGS, RNAi, overexpression, and CRISPR/Cas9 systems, or a combination of these, has been established. Application of these tools has proved most informative in strawberry studies [44, 65, 144–146], and is likely to facilitate further exploration of mechanisms of strawberry fruit ripening mediated by ABA. This makes strawberry a model platform for guiding the exploration of ripening of other fruits, especially non-climacteric fruits, to reveal their ripening mechanisms.
